# Microbe Profile: Bacteriophage ϕ6: a model for segmented RNA viruses and the evolutionary consequences of viral ‘sex’

**DOI:** 10.1099/mic.0.001467

**Published:** 2024-07-24

**Authors:** Paul E. Turner, Lin Chao

**Affiliations:** 1 Department of Ecology and Evolutionary Biology, Yale University, New Haven, CT 06511, USA; 2 Program in Microbiology, Yale School of Medicine, New Haven, CT 06520, USA; 3 Center for Phage Biology and Therapy, Yale University, New Haven, CT 06511, USA; 4 School of Biological Sciences, University of California San Diego, La Jolla, CA 92093, USA

**Keywords:** adaptation, bacteria, evolution, experimental evolution, *Pseudomonas*, phage

## Abstract

Bacteriophage ϕ6 is a segmented dsRNA virus with a lipid envelope, which are unusual traits in bacterial viruses but common in eukaryotic viruses. This uniqueness allowed ϕ6 and its *Pseudomonad* hosts to serve as a molecular model for RNA genetics, mutation, replication, packaging, and reassortment in both bacterial and eukaryotic viruses. However, an additional uniqueness of ϕ6, created by its high mutation rate, was its use as an experimental system to study key questions such as the evolution of sex (segment reassortment), host–pathogen interactions, mutational load, rates of adaptation, genetic and phenotypic complexity, and game theory.

## Graphical abstract

Phage ϕ6 is a model for segmented RNA viruses, and for testing evolutionary biology. (a) Phage ϕ6 attaches to type-IV pili of susceptible host bacteria and selects for phage-resistance via altered/deleted pili, causing a ‘trade-off’ that should reduce pathogenicity (leaf adherence and plant invasion) of *Pseudomonad* bacteria in the wild. (b) Muller’s ratchet predicts that asexual populations of small size should accrue harmful mutations that reduce mean fitness over time, depicted as rightward shift in a hypothetical histogram (blue bars) of individuals harbouring greater average genetic load. Whereas ϕ6 studies showed segment reassortment (viral sex) can create hybrids with fewer mutations (horizontal red bars on RNA segments) than those carried by co-infecting ‘parent’ viruses, causing leftward shift of histogram, and reduced average genetic load over time. (c) Wild-type phage (black) is advantaged in particle production, relative to a cheater phage (red). But frequent co-infection can favour selection for cheater variants of ϕ6 that outcompete the wild-type; overall productivity decreases but cheaters are over-represented among the viral progeny compared to their production when infecting cells alone. This result is consistent with the Prisoner’s Dilemma outcome in evolutionary game theory, which shows a cheating strategy (red line depicting cheater fitness) can invade a population at any initial starting frequency (exceeding horizontal black line for wild-type fitness defined as 1.0) despite causing mean fitness to decline through time (red arrow pointing down and to the right as cheaters approach fixation when frequency equals 1.0).

## Taxonomy

Realm: Riboviria; kingdom: Orthornavirae; phylum: Duplornaviricota; class: Vidaverviricetes; order: Mindivirales; family: *Cystoviridae*; genus: *Cystovirus*; species: *Cystovirus phi6*.

## Properties

The bacteriophage (phage) ϕ6 virion consists of an outer lipid envelope surrounding a nucleocapsid shell and an icosahedral capsid core, which contains three double-stranded RNA (dsRNA) segments; these are unusual properties of known phages, found only in ϕ6 and its cystovirus relatives [1]. Phage ϕ6 was first isolated by Vidaver *et al.* [2] from bean straw, and propagated in the laboratory on various *Pseudomonas syringae* strains. The virus adsorbs to host cells via a type-IV pilus; when retracted this allows virus membrane fusion with the bacterial outer-membrane, placing the viral nucleocapsid in the periplasmic space, whereupon host-cell entry permits copies of the three dsRNA segments to be transcribed [1]. The phage is strictly lytic and capable of infecting various *Pseudomonad* species, where changes in its host range can occur minimally via point mutations. Host-range mutations are observed at a frequency of 3×10^−4^, which reflects the high mutation rate of RNA viruses [3]. Typical laboratory propagation is on *Pseudomonas syringae* pathovar *phaseolicola* strain HB10Y, cultured at 25 °C. The multilayered structure of phage ϕ6 particles is well described, including functions and molecular weights of basic structural proteins comprising the inner procapsid layer, matrix layer consisting of shell protein, and outer layer, which is the cell envelope bilipid membrane layer with phage membrane proteins, lytic endopeptidase and binding protein [1].

## Genome

The ϕ6 genome is roughly 13.5 kb, split into three dsRNA segments named by their sizes: large (L; ~6.4 kb), medium (M; ~4.1 kb) and small (S; ~2.9 kb). The genome encodes 13 viral proteins, where each segment contains multiple ORFs flanked by 5′ and 3′ UTRs. The genome is organized such that genes for structural proteins of the virus capsid occur on L; genes for membrane attachment proteins and host specificity on M; and genes for nucleocapsid shell, major membrane protein, lytic endopeptidase (lysin) and membrane-assembly nonstructural protein on S [1]. During ϕ6 intracellular reproduction, viral polymerases transcribe infecting dsRNA segments into (+)RNA molecules, which act both as templates for protein synthesis and consist of the genome forms incorporated into nascent particles within the host cell [4]. The procapsid first displays only the binding site that permits S (+)RNA segment to be packaged, followed by conformational changes that reveal binding sites for M (+)RNA, and finally L (+)RNA segments to package. Following segment entry, viral polymerases convert the (+)RNA segments into dsRNA through a single round of (−)RNA synthesis. If (+)RNA strands are produced solely by the infecting dsRNA segments, only one or a few templates would produce progeny and phi6 replication acts as a stamping machine [5]. This contrasts with the semi-conservative replication of DNA in which all templates make progeny. A stamping machine would produce a lower load of deleterious mutations than semi-conservative replication when the mutation rate is high [5], and the possibility of stamping machine replication in cystoviruses merits additional study. Further morphogenesis includes acquisition of the outer envelope, leading to production of fully infectious ϕ6 particles.

## Phylogeny

Phylogenomic reconstruction of the evolution of RNA viruses has been attempted using the only gene these viruses share in common: RNA-dependent RNA polymerase (RdRp) [6]. However, obtaining strongly supported phylogenies for RdRps is challenging due to the extensive sequence divergence in these genes, aside from several conserved motifs that are required for polymerase activity. Nevertheless, Wolf *et al*. [6] used thousands of available RdRp sequences to infer virus relationships, producing an RNA-virus phylogenetic tree with well-resolved topology in which the tree splits into five major branches, each containing a substantial diversity of viruses. Phage ϕ6 and its relatives in the cystoviruses were placed in branch 4 of the tree that consisted solely of dsRNA viruses. This result inferred that cystoviruses shared relatedness to reoviruses, totiviruses and additional families of viruses that infect eukaryote host species. The tree topology was compatible with the monophyly of RdRps, and was used to infer the origin of eukaryotic RNA viruses from a prokaryotic RNA virus ancestor shared with leviviruses [6]. The concept of close phylogenetic relatedness of cystoviruses and reoviruses seems corroborated by similar structural features of their icosahedral capsids, as well as evolution of certain clade-specific genes [6].

## Experimental evolution using ϕ6

ϕ6 has also been used for a variety of studies in experimental evolution, largely driven by the virus exhibiting two evolutionary mechanisms that were arguably the most important for generating genetic variation: a high mutation rate and segment reassortment (a form of viral sex). Below we provide summaries of some key experimental-evolution studies using ϕ6.

## Mutation accumulation in asexual populations

The high mutation rate of ϕ6 can lead to the accumulation of deleterious mutations (increase mutational load) when the virus population is numerically small in size; here, the effects of genetic drift should overwhelm natural selection’s ability to eliminate deleterious mutations, thus causing the fitness of the virus population to decline over successive generations. Chao [7] used ϕ6 in the laboratory to demonstrate this effect of mutational load for viruses propagated under small population sizes, consistent with the classic idea (Muller’s ratchet) that increased mutational load is problematic in asexual populations of small size. However, the ratchet can be reversed if reassortment of segments during co-infection can produce hybrids (reassortants) with fewer mutations than were present in the co-infecting parent viruses. Indeed, subsequent experiments showed that reassortment can be advantageous in ϕ6, because co-infection between low-fitness viruses can yield progeny of higher fitness than the parents.

## Mutational spectrum and biological complexity

The increase of the mutational load via Muller’s ratchet revealed one aspect of the mutational spectrum of ϕ6; the abundance of deleterious mutations exceeds that of beneficial ones. Subsequent ϕ6 experiments revealed additionally that the spectrum consisted of more mutations of small fitness effect than ones of large effect [8], which confirmed predictions of classic population-genetics theory that argue adaptive evolution necessarily proceeds through substitutions of small effect because they are relatively more numerous.

## Evolutionary consequences of virus co-infection

Additional studies have used ϕ6 to test hypotheses on the evolutionary advantages of sex (genetic mixis) in populations of large size. Turner and Chao [9] controlled whether reassortment was common versus rare in experimentally evolved ϕ6 lineages, by mixing viruses and host cells to create high versus low rates of co-infection. Unexpectedly, these experiments showed that rates of virus adaptation were fastest when co-infection was rare, counter to generalized predictions for the evolutionary advantages of sex. This puzzling outcome was later revealed as a consequence of co-infection that overwhelmed its predicted benefits; frequent co-infection selected for ϕ6 viruses evolved to parasitize other phage genotypes during intra-cellular replication, which exacted a tradeoff that caused these virus ‘cheaters’ to be less fit at exploiting the host cell on their own. This tradeoff could be modelled as the Prisoner’s Dilemma of game theory, which predicts cheating can evolve in a biological system despite reduced overall fitness of the population when cheaters displace the non-cheater ancestor [10]. The evolution of ultra-parasitic phages unable to reproduce in the absence of other (helper) phages would correspond to the snow-drift game, but this was not observed. One possible explanation is the number of phages co-infecting the same host cell remained small (two–three phages maximum), owing to an exclusion mechanism that limits the number of phages capable of co-infecting a single host [9].

## Open questions

- Because cystoviruses are the only known family of phages with envelopes and segmented genomes, does this rarity indicate phages are constrained when evolving and maintaining such properties?
- How is ϕ6 naturally capable of infecting a wide variety of Pseudomonads, with minimal genetic changes (point mutations) governing even-broader host-range use?
- Because ϕ6 and its cystovirus relatives are unique among known phages as enveloped segmented viruses, can their inclusion in RNA virus phylogenetics help resolve ancient relatedness between viruses of prokaryotes and eukaryotes?
- How can ϕ6 be harnessed further as a model to explore the maintenance of traits in biological systems that experience high mutation rates, such as RNA viruses that may accumulate deleterious mutations when evolving under frequent population bottlenecks?

## Abbreviations

dsRNA: double-stranded RNA
kb: kilobase
ORF: open-reading frame
(−)RNA: negative-sense RNA
(+)RNA: positive-sense RNA
UTR: untranslated region
